# Clinical characteristics of community-acquired pneumonia due to *Moraxella catarrhalis* in adults: a retrospective single-centre study

**DOI:** 10.1186/s12879-020-05564-9

**Published:** 2020-11-10

**Authors:** Jun Hirai, Takeshi Kinjo, Tomomi Koga, Shusaku Haranaga, Eiji Motonaga, Jiro Fujita

**Affiliations:** 1 Department of Internal Medicine, Okinawa Miyako Hospital, Okinawa, Japan; 2 Department of Infectious, Respiratory and Digestive Medicine, Graduate School of Medicine, University of the Ryukyus, 207 Uehara, Nishihara, Okinawa, 903-0215 Japan; 3 Department of Radiology, Graduate School of Medicine, University of the Ryukyus, Okinawa, Japan; 4 Department of General Medicine, Okinawa Miyako Hospital, Okinawa, Japan

**Keywords:** *Moraxella catarrhalis*, *Streptococcus pneumoniae*, Community-acquired pneumonia, Adult, Winter, Asthma, Bronchiectasis, Influenza virus, Bronchopneumonia pattern, Gram stain

## Abstract

**Background:**

Although *Moraxella catarrhalis* (*M. catarrhalis*) is a common cause of community-acquired pneumonia (CAP), studies investigating clinical manifestations of CAP due to *M. catarrhalis* (MC-CAP) in adults are limited. Since *S. pneumoniae* is the leading cause of CAP globally, it is important to distinguish between MC-CAP and CAP due to *S. pneumoniae* (SP-CAP) in clinical practice. However, no past study compared clinical characteristics of MC-CAP and SP-CAP by statistical analysis. We aimed to clarify the clinical characteristics of MC-CAP by comparing those of SP-CAP, as well as the utility of sputum Gram staining.

**Methods:**

This retrospective study screened CAP patients aged over 20 years visiting or admitted to Okinawa Miyako Hospital between May 2013 and April 2018. Among these, we included patients whom either *M. catarrhalis* alone or *S. pneumoniae* alone was isolated from their sputum by bacterial cultures.

**Results:**

We identified 134 MC-CAP and 130 SP-CAP patients. Although seasonality was not observed in SP-CAP, almost half of MC-CAP patients were admitted in the winter. Compared to those with SP-CAP, MC-CAP patients were older (*p* < 0.01) and more likely to have underlying pulmonary diseases such as asthma and bronchiectasis (*p* < 0.01). Approximately half of asthmatic MC-CAP and SP-CAP patients had asthma attacks. Although winter is an influenza season in Japan, co-infection with influenza virus was less common in MC-CAP compared to SP-CAP patients (3% vs. 15%, *p* < 0.01). Bronchopneumonia patterns on X-ray, as well as bronchial wall thickening, bilateral distribution, and segmental pattern on CT were more common in MC-CAP patients than in SP-CAP patients (*p* < 0.01). Sputum Gram stain was highly useful method for the diagnosis in both MC-CAP and SP-CAP (78.4% vs. 89.2%), and penicillins were most frequently chosen as an initial treatment for both pneumonias.

**Conclusions:**

This is the first study to show that MC-CAP occurred in older people compared to SP-CAP, influenza virus co-infection was less common in MC-CAP than SP-CAP, and that MC-CAP frequently caused asthma attacks. Gram stain contributed for the appropriate treatment, resulting in conserving broad-spectrum antibiotics such as cephalosporins and fluoroquinolones in both MC-CAP and SP-CAP patients.

## Background

Although *Moraxella catarrhalis* is a common bacterial cause of community-acquired pneumonia (CAP) [1, 2], detailed information regarding the clinical features of CAP due to *M. catarrhalis* (MC-CAP) in adults is limited. It is generally considered that the incidence of MC-CAP is high in the elderly and persons with chronic pulmonary diseases in the winter season; however, this information is based on descriptive studies, most of which date back to the 1980s, and the number of patients included in these studies was relatively small [3–5]. There have been only two studies on MC-CAP published with a sample size of > 100 patients [4, 5]. In addition, previous studies have not excluded patients co-infected with other respiratory bacteria, so the clinical features of MC-CAP isolated only *M. catarrhalis* by bacterial culture are unclear [4, 5]. In terms of radiological features of MC-CAP, Okada et al. [6] assessed pulmonary computed tomography (CT) findings in patients with acute *M. catarrhalis* pulmonary infection. However, 75 of 109 patients (68.8%) had nosocomial infection, and radiological findings of MC-CAP were not specifically described. Additionally, although *M. catarrhalis* causes acute exacerbation of chronic obstructive pulmonary disease (COPD) [7, 8], none of the previous studies have investigated the relationship between asthma attacks and MC-CAP. Moreover, while it is well known that antecedent influenza virus infection can induce secondary *Streptococcus pneumoniae* pneumonia [9], the association between influenza virus infection and MC-CAP in adults remains unknown. Furthermore, no studies have made statistical comparisons of the clinical features of MC-CAP and CAP due to *S. pneumoniae* (SP-CAP) in adults. Because *S. pneumoniae* is the leading cause of CAP globally, it is useful for physicians to be able to distinguish between MC-CAP and SP-CAP in clinical practice.

Gram stain examinations are easy, rapid, and useful for identifying causative bacteria; however, it is recently abandoned in the US and European countries partially for non-scientific reasons such as legal and economic pressures [10]. In our facility, attending physicians perform Gram staining themselves and choose an initial antibiotic based on the result. Gram stain-guided choice of narrow-spectrum antibiotics, rather than empirical use of broad-spectrum antibiotics, can inhibit the emergence of drug-resistant bacteria. Therefore, evaluation of the utility of Gram staining is especially important in the post-antibiotic era.

In the current study, the clinical characteristics of MC-CAP were evaluated by comparison with those of SP-CAP. Additionally, diagnostic utility of Gram staining and choosing antibiotics based on Gram stain result were also evaluated.

## Methods

### Patients and study design

In this retrospective observational study, we initially screened consecutive adult CAP patients aged over 20 years, in whom pneumonia had developed during daily community living, who visited or were admitted to Okinawa Miyako Hospital (an acute care hospital on Miyako Island, Okinawa, Japan) between May 2013 and April 2018. We extracted patients that had either *M. catarrhalis* alone or *S. pneumoniae* alone, as determined by bacterial culture from their expectorated sputum with grades P1, P2, or P3 as classified by Miller and Jones [11]. Thus, patients co-infected with other respiratory bacteria were excluded in this study.

### Definition of CAP and pneumonia diagnosis

The diagnosis of CAP was based on the presence of clinical lower respiratory symptoms such as cough, expectorated sputum, and dyspnoea in addition to fever (≥37 °C) combined with new pulmonary infiltrates on chest X-ray [12]. Patients were excluded if they met the following conditions: 1) they were under corticosteroids and/or other immunosuppressive therapy; 2) antibiotic therapy was initiated before collecting sputum and blood for bacterial culture; 3) presence of other diseases that complicate respiration and make it difficult to accurately diagnose pneumonia, such as acute heart failure; and 4) residing in a nursing home or a long-term care facility.

### Evaluation severity of CAP

The CURB-65 score recommended by the British Thoracic Society was used to evaluate the severity of CAP [13], and the Quick Sepsis-related Organ Failure Assessment (qSOFA) score was used to screen for sepsis [14].

### Data collection

Data were retrospectively collected from medical records. Two sets of blood cultures were obtained from every patient before administrating antimicrobial agents. Additionally, a rapid influenza diagnostic test was performed on all patients upon admission. COPD exacerbation and asthma attack were defined as conditions presenting with shortness of breath, wheezing, and in-hospital administration of bronchodilator or corticosteroids. Chest X-ray and CT were evaluated by two physicians (one radiologist and one pulmonologist). On chest X-ray, bronchopneumonia pattern includes multiple areas of small nodular and/or patchy consolidation without air bronchogram. While, lobar pneumonia, also known non-segmental pneumonia, pattern shows a solitary, peripheral focus of dense opacity with air bronchogram.

### Sputum evaluation, intubation, and antimicrobial susceptibility

Gram stain of sputum was performed in all patients upon admission. Polymicrobial pattern was defined as the presence of many different bacteria without a predominant bacterium upon Gram stain. The presumptive bacteria and their morphotypes were as follows: gram-positive, lancet-shaped diplococci for *S. pneumoniae*, and gram-negative diplococci for *M. catarrhalis*. Sputum specimens were cultured on sheep blood agar and incubated at 37 °C in 5% CO_2_ for 24–48 h. The phenotypic identification of isolates and antibiotic susceptibility testing was performed by VITEK 2 (bioMérieux, Marcy-l’Étoile, France). The breakpoint for susceptibility testing was based on Clinical Laboratory Standards Institute (CLSI) M100-S22.

### Statistical analysis

We used Pearson’s χ^2^ or Fisher’s exact test and the Mann-Whitney U test to compare characteristics of MC-CAP and SP-CAP patients for categorical and continuous variables, respectively. A *p*-value of < 0.05 was considered statistically significant. All data were analysed using R version 2.13.1 (R Foundation for Statistical Computing, Vienna, Austria).

### Ethical approval

The Institutional Ethics Committee of Okinawa Miyako Hospital approved this study (approval number 18 M005). The need for informed consent from each patient for inclusion in this study was waived because this study was retrospective, and there were no study-related interventions.

## Results

### Clinical characteristics of MC-CAP and SP-CAP patients

**Fig. 1:**
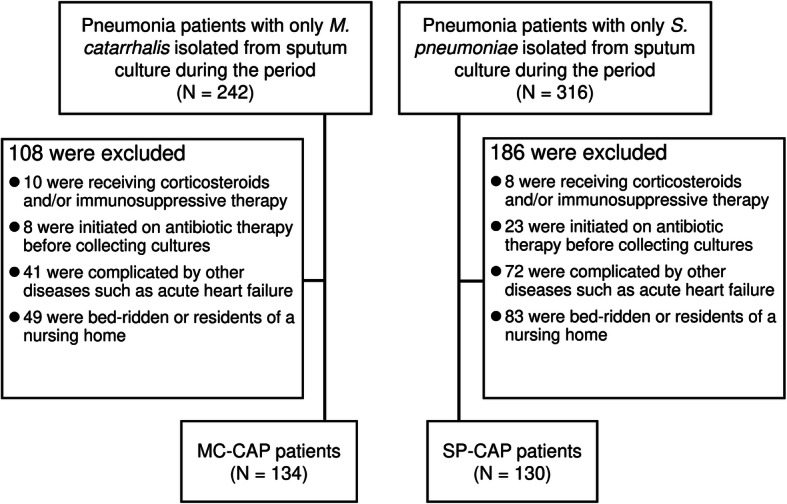
Flow chart showing the selection for MC-CAP and SP-CAP patients for this study. Eligible patients were further selected by applying the exclusion criteria described in the Materials and Methods section

**Fig. 2:**
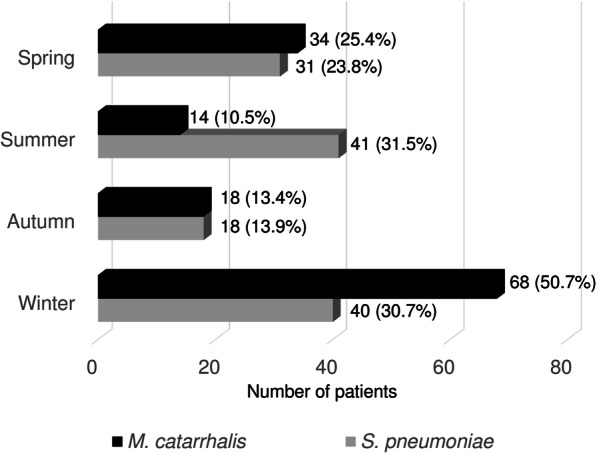
Seasonality of MC-CAP and SP-CAP. Spring: March to May; Summer: June to August; Fall: September to November; Winter: December to February

**Fig. 3:**
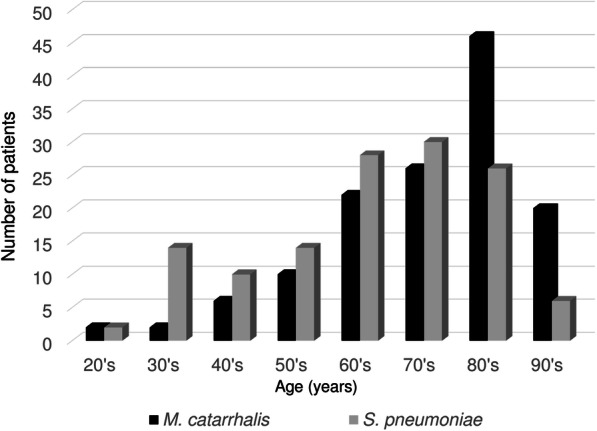
Age distribution for MC-CAP and SP-CAP patients

During the study period, 134 and 130 patients were diagnosed as MC-CAP and SP-CAP, respectively (Fig. 1). Although seasonality was not observed in SP-CAP, almost half (50.7%) of MC-CAP patients were admitted in winter (Fig. 2). As shown in Fig. 3, both conditions frequently occurred in the elderly, with the greatest number of MC-CAP and SP-CAP patients being in their 80s and 70s, respectively.

Patients’ backgrounds and clinical characteristics are shown in Table 1. The mean age of MC-CAP patients was significantly higher than that of SP-CAP patients (75.2 vs. 66.2 years; *p* < 0.01). There were no statistically significant differences in the sex and smoking history between the two groups. Body mass index in MC-CAP patients was higher than in SP-CAP patients (23.6 vs. 22.4; *p* < 0.05). MC-CAP patients were more likely than those with SP-CAP to have underlying pulmonary diseases such as asthma (*p* < 0.01) and bronchiectasis (*p* < 0.01), and were more likely to be using home oxygen therapy (*p* < 0.01). In addition, chronic heart failure (*p* < 0.01) and chronic kidney disease (*p* < 0.01) were more frequent in MC-CAP patients. A relatively small proportion of each group had severe pneumonia according to their CURB-65 score, and only 3.7% of MC-CAP and 6.2% of SP-CAP patients had a positive qSOFA score (≥2). In terms of clinical manifestations and laboratory data, the proportions of high fever (*p* < 0.01), systolic blood pressure ≤ 100 mmHg (*p* < 0.05), shaking chill (*p* < 0.01), elevated white blood cell counts (*p* < 0.05), and a positive influenza rapid test (*p* < 0.01) were significantly higher in patients with SP-CAP than in those with MC-CAP. There was no significant difference in the frequency of COPD exacerbation between the two groups. Approximately half of the patients with previously diagnosed asthma in each group experienced asthma attacks. Bacteremic pneumonia occurred in only 0.7 and 1.5% of MC-CAP and SP-CAP patients, respectively (*p* = 0.54).

**Table 1:** Baseline characteristics of MC-CAP and SP-CAP patients

	MC-CAP (%)	SP-CAP (%)	***P*** value^**a**^
Number of patients	134	130	
**Background**			
Age: mean ± SD	75.2 ± 15.6	66.2 ± 17.4	< 0.01
Gender (M/F)	72/62	64/66	0.46
Body mass index^b^, mean ± SD	23.6 ± 5.6	22.4 ± 3.5	< 0.05
Ever smoker	55 (41)	42 (32.3)	0.14
**Underlying pulmonary disease**			
Asthma	51 (38.1)	25 (19.2)	< 0.01
Bronchiectasis	56 (41.8)	22 (16.9)	< 0.01
Chronic obstructive pulmonary disease (COPD)	35 (26.1)	26 (20)	0.24
Interstitial pneumonia	6 (4.5)	1 (0.8)	0.06
Lung cancer	6 (4.5)	4 (3.1)	0.55
Old tuberculosis	15 (11.2)	10 (7.7)	0.33
Under home oxygen therapy	16 (11.9)	4 (3.1)	< 0.01
Usage continuous positive airway pressure therapy	6 (4.5)	1 (0.8)	0.06
**Systemic underlying disease**			
Cerebrovascular disease	7 (5.2)	4 (3.1)	0.38
Chronic heart failure	41 (30.6)	18 (13.8)	< 0.01
Chronic kidney disease	15 (11.2)	3 (2.3)	< 0.01
Collagen disease	3 (2.2)	0 (0)	0.08
Diabetes mellitus	23 (17.2)	14 (10.8)	0.13
Hypertension	64 (47.8)	48 (36.9)	0.07
Malignancy	15 (11.2)	8 (6.2)	0.14
**Severity**			
CURB-65			
Mild	96 (71.6)	103 (79.2)	0.15
Moderate	32 (23.9)	22 (16.9)	0.16
Severe	6 (4.5)	5 (3.8)	0.79
qSOFA			
≥ 2	5 (3.7)	8 (6.2)	0.36
**Clinical manifestation**			
High fever (≥38 °C)	50 (37.3)	80 (61.5)	< 0.01
Systolic blood pressure ≤ 100 mmHg	3 (2.2)	11 (8.5)	< 0.05
Shaking chill	8 (6)	26 (20)	< 0.01
COPD exacerbation	10/35 (28.6)	5/26 (19.2)	0.4
Asthma attack	24/51 (47)	13/25 (52)	0.68
**Laboratory data**			
White blood cell^c^, mean ± SD	11,451 ± 4556	12,543 ± 5118	< 0.05
C-reactive protein^d^, mean ± SD	8.2 ± 7.4	9.1 ± 7.6	0.17
Serum albumin^e^, mean ± SD	3.6 ± 0.5	3.7 ± 0.5	0.21
Positive for influenza virus rapid test	4 (3)	19 (14.6)	< 0.01
**Blood culture**			
Positive	1 (0.7)	2 (1.5)	0.54
**Initial treatment**			
Penicillin (ABPC, ABPC/SBT, AMPC, or AMPC/CVA)	70 (52.2)	71 (54.6)	0.69
Cephalosporin (CTM, CTRX, CTX, or CMZ)	48 (35.8)	51 (39.2)	0.57
Macrolides (AZM)	8 (6)	6 (4.6)	0.62
Tetracyclines (MINO)	5 (3.7)	0 (0)	< 0.05
Fluoroquinolones (LVFX)	2 (1.5)	1 (0.8)	0.57
**Outcome**			
Hospitalised patients	79 (59)	76 (58.4)	0.93
Length of antibiotic treatment, mean ± SD	6.8 ± 2.2	7.1 ± 2.3	0.17
Length of hospital stay, mean ± SD	9.2 ± 4.7	8.6 ± 3.0	0.19
In-hospital mortality	0	0	–

*Abbreviations*: *COPD* chronic obstructive pulmonary disease, *SD* standard deviation, *ABPC* ampicillin, *ABPC/SBT* ampicillin sulbactam, *AMPC* amoxicillin, *AMPC/CVA* amoxicillin clavulanate, *CTM* cefotiam, *CTRX* ceftriaxone, *CTX* cefotaxime, *CMZ* cefmetazole, *AZM* azithromycin, *MINO* minocycline, *LVFX* levofloxacin

^a^Statistical differences between MC-CAP and SP-CAP were evaluated by Pearson’s χ2 or Fisher’s exact test for categorical variables and Mann-Whitney U test for continuous variables

^b^Number of patients was 108 in MC-CAP and 93 in SP-CAP

^c^Number of patients was 118 in MC-CAP and 113 in SP-CAP

^d^Number of patients was 117 in MC-CAP and 109 in SP-CAP

^e^Number of patients was 98 in MC-CAP and 91 in SP-CAP

All *M. catarrhalis* isolates produced beta-lactamase, while all *S. pneumoniae* isolates were susceptible to penicillin. Most MC-CAP and SP-CAP patients were treated with penicillins or cephalosporins, and there were no significant differences in the selection of initial antibiotics other than tetracyclines among the two groups. Nearly two-thirds of patients in both groups were hospitalised, and there was no statistically significant difference in the duration of antibiotic treatment or the length of hospital stay between the two groups. All patients with MC-CAP or SP-CAP received oral and intravenous antibiotics targeting susceptible isolates, and there were no deaths in either group.

### Radiological findings

Chest radiography and CT findings in MC-CAP and SP-CAP patients are shown in Table 2. The bronchopneumonia pattern on chest X-ray was similar in both groups. However, this pattern was more frequently observed in MC-CAP patients than in those with SP-CAP (*p* < 0.01). Among the 18 MC-CAP patients and 23 SP-CAP patients who had chest CT scans, bronchial wall thickening (66.7 vs. 26.1%; *p* < 0.01), bilateral distribution (88.9 vs. 47.8%; *p* < 0.01), and segmental pattern (100 vs. 69.5%; *p* < 0.01) were more common in MC-CAP patients. Of the SP-CAP patients, 30.4% had a pleural effusion while none of the MC-CAP patients had a pleural effusion (*p* < 0.01).

**Table 2:** Chest X-ray and CT findings in MC-CAP and SP-CAP patients

	MC-CAP (%)	SP-CAP (%)	***P*** value^**a**^
**Chest X-ray**			
Number of patients	134	130	
Bronchopneumonia pattern	127 (94.8)	84 (64.6)	< 0.01
Lobar pneumonia pattern	7 (5.2)	46 (35.4)	< 0.01
**Chest CT**			
Number of patients	18	23	
**Findings**			
Consolidation	6 (33.3)	19 (82.6)	< 0.01
Air bronchogram	3 (16.7)	17 (73.9)	< 0.01
Ground glass opacities	7 (38.9)	20 (87)	< 0.01
Bronchial wall thickenings	12 (66.7)	6 (26.1)	< 0.01
Centrilobular nodules	12 (66.7)	20 (87)	0.11
Nodules (5–30 mm)	7 (38.9)	17 (73.9)	< 0.05
Pleural effusion	0 (0)	7 (30.4)	< 0.01
Lymph node enlargement (over 1 cm diameter)	2 (11.2)	10 (43.5)	< 0.05
**Distribution**			
Unilateral	2 (11.1)	12 (52.2)	< 0.01
Bilateral	16 (88.9)	11 (47.8)	< 0.01
Segmental pattern	18 (100)	16 (69.5)	< 0.01
Non-segmental pattern	0 (0)	7 (30.4)	< 0.01

^a^Statistical differences between MC-CAP and SP-CAP were evaluated by Pearson’s χ^2^ or Fisher’s exact test

### Gram stain and initial treatment

The sensitivity of the Gram stain for MC-CAP diagnosis was significantly lower than that for *S. pneumoniae* (78.4 vs. 89.2%; *p* < 0.05). *M. catarrhalis* was most frequently misidentified as *Haemophilus influenzae* (8.2%) or *S. pneumoniae* (6%), while *S. pneumoniae* was most frequently misidentified as *M. catarrhalis* (3.1%) (Table 3).

**Table 3:** Microorganisms assumed by Gram stain

	MC-CAP (%)	SP-CAP (%)
*M. catarrhalis*	105 (78.4)	4 (3.1)
*H. influenzae*	11 (8.2)	0
*S. pneumoniae*	8 (6)	116 (89.2)
Polymicrobial	9 (6.7)	9 (6.9)
None	1 (0.7)	1 (0.8)

## Discussion

As mentioned earlier, current knowledge about the clinical features of MC-CAP in adults is based on old studies, and most of these studies are descriptive [1–5]. In the current study, we used statistical inference to compare the clinical characteristics of CAP caused by *M. catarrhalis* infections with those with CAP cause by *S. pneumoniae* infections. In addition to reconfirming the previously known characteristics of MC-CAP, we found for the first time that co-infection with influenza virus was less common in MC-CAP patients compared to those with SP-CAP, even though half of MC-CAP patients were admitted during the influenza season. Furthermore, both MC-CAP and SP-CAP frequently caused asthma attacks.

As shown in previous studies [1, 3, 15], MC-CAP patients in the present study were frequently admitted in winter. The reason for this pattern is unknown. Some investigators have described an association with a preceding or concurrent viral infection [16, 17], but our data show that influenza virus infection is not common in MC-CAP. Infections with other respiratory viruses were not examined in this study; therefore, any associations with other respiratory viruses remain undetermined. Borges et al. [18] showed that the occurrence of MC-CAP in children in tropical regions was positively associated with low humidity and negatively associated with air temperature and sunshine, suggesting that climatic conditions might account for the seasonality of MC-CAP. Further studies are needed to address this question.

MC-CAP was more common in the elderly and more likely to complicate underlying pulmonary diseases in the present study, a result that is consistent with previous studies [19, 20]. Elderly patients’ propensity to develop MC-CAP might be explained by the asymptomatic carriage rate of *M. catarrhalis*. The carriage rate in those under 60 years old (5%) increases to 25% in those over 60 years old [19]. Past studies have shown that widespread use of pneumococcal vaccines increases the prevalence of *M. catarrhalis* colonisation in the respiratory tract [21, 22]. Therefore, we expect to see an increase in the incidence of MC-CAP, particularly among the elderly, as the global population ages and pneumococcal vaccine coverage increases. For this reason, we must pay attention to the epidemiological trends of MC-CAP in elderly patients.

To the best of our knowledge, no previous studies have investigated the rate of asthma attacks in MC-CAP patients. As *M. catarrhalis* adheres to mucosal surfaces and induces an inflammatory response in bronchial epithelial cells, it is not surprising that it can trigger an asthma attack [23, 24]. In addition, Alnahas et al. [25] demonstrated that *M. catarrhalis* infection induced IL-17 and TNF-α production in the airways and triggered asthma attacks in murine models. Thus, additional clinical studies are needed to clarify the relationship between asthma attacks and *M. catarrhalis* infection.

Studies of the radiological features of MC-CAP are limited. Additionally, no past studies have compared the radiological findings of MC-CAP and SP-CAP. Okada et al. [6] investigated 109 CT scans conducted on patients with *M. catarrhalis* pneumonia (only 34 of 109 patients had CAP) and found that the most common radiological findings were ground glass opacities (91%) followed by bronchial wall thickening (78%), centrilobular nodules (73%), and consolidation (49%). These findings were similar to our findings, suggesting that these findings are characteristic of *M. catarrhalis* respiratory infection regardless of the pneumonia classification as CAP or hospital-acquired pneumonia.

Gram stain examination is a simple and rapid diagnostic tool for the presumptive identification of causative bacteria in patients with CAP. Its diagnostic usefulness in the selection of appropriate antibiotics in clinical practice has been investigated in several previous studies [26, 27]. In the current study, the sensitivity of sputum Gram stain for MC-CAP diagnosis was lower than that for SP-CAP; however, the rate was relatively high. Fukuyama et al. [26] reported that the sensitivity of sputum Gram stain for MC-CAP diagnosis was higher than that for SP-CAP diagnosis (85.0% vs. 63.1%), although the number of patients included the study was small (20 and 76 patients with MC-CAP and SP-CAP, respectively). Our data show that Gram stain can guide the appropriate use of antibiotics; more than half the MC-CAP and SP-CAP patients were treated with penicillins. Drug-resistant bacteria are an increasingly serious problem worldwide, and we need conserve existing antibiotic drugs, particularly broad-spectrum antimicrobial agents. By having attending physicians perform a Gram stain, we could treat pneumonia patients with targeted, narrow-spectrum antibiotics in this study, rather than empirical, broad-spectrum antibiotics such as cephalosporins and quinolones.

The mortality rate of MC-CAP patients in this study was 0% even though previous studies have revealed rates ranging from 5 to 21.4% [1, 4, 6, 28]. In fact, most patients included in this study were not classified as having severe pneumonia according to the CURB-65 score. This might be because we excluded patients who were bedridden, residing in a nursing home, receiving an immunosuppressive therapy, or had other diseases complicating respiration, such as acute heart failure. We also excluded patients with concurrent bacterial co-infection; excluding these patients might affect the mortality. In addition, Gram stain-guided appropriate selection of antibiotics might reduce mortality. It is noteworthy that zero mortality was achieved with a penicillin-centred choice of antibiotics. Since Gram staining is generally performed by attending physicians for all patients in most hospitals in Okinawa [10], this study reconfirms the validity of Gram stain-guided prompt decision making in clinical practice.

The present study has several strengths. Firstly, the number of MC-CAP patients included in this study was larger than that in the previous studies. Additionally, we excluded MC-CAP patients co-infected with other respiratory bacteria; therefore, our study population was appropriate for evaluating the characteristics of MC-CAP. Secondly, rather than performing a descriptive study, we evaluated the characteristics of MC-CAP by comparing them with those of SP-CAP. Thirdly, our study is the first to determine the co-infection rate of MC-CAP with influenza virus as well as the rate of asthma attacks among MC-CAP patients.

Our study has certain limitations. Firstly, it was a retrospective study conducted in a single centre. Secondly, it is possible that some MC-CAP patients had co-infection with additional unidentified atypical pathogens and viruses. However, co-infection with atypical bacteria and respiratory viruses was not common in MC-CAP [29]; therefore, this limitation may not significantly affect our results. Finally, we did not perform chest CT examinations in all pneumonia patients. However, only one study in the literature examined the characteristics of chest CT findings in MC-CAP patients, and our results were similar to the findings of that study [6].

## Conclusions

We were able to elucidate the clinical features of MC-CAP by performing a statistical comparison with SP-CAP. We revealed for the first time that co-infection with influenza virus was less common in MC-CAP patients, and that MC-CAP caused asthma attack in similar frequency to SP-CAP. Additionally, we showed that Gram staining contributes to the appropriateness of treatments, resulting in reducing broad-spectrum antibiotic use and lowering mortality. Physicians should be aware of MC-CAP because it will likely increase in prevalence with the proliferation of pneumococcal vaccines as the global population ages.

## Data Availability

The datasets used and/or analysed during the current study are available from the corresponding author on reasonable request.

## Abbreviations

CAP: Community-acquired pneumonia
MC-CAP: Community-acquired pneumonia (CAP) due to *M. catarrhalis*
SP-CAP: Community-acquired pneumonia due to *S. pneumoniae*
CT: Computed tomography
COPD: Chronic obstructive pulmonary disease
qSOFA: Quick sepsis-related organ failure assessment
SD: Standard deviation
ABPC: Ampicillin
ABPC/SBT: Ampicillin sulbactam
AMPC: Amoxicillin
AMPC/CVA: Amoxicillin clavulanate
CTM: Cefotiam
CTRX: Ceftriaxone
CTX: Cefotaxime
CMZ: Cefmetazole
AZM: Azithromycin
MINO: Minocycline
LVFX: Levofloxacin
